# Clinicopathological and Treatment Patterns of Combined Small-Cell Lung Carcinoma with Future Insight to Treatment: A Population-Based Study

**DOI:** 10.3390/jcm12030991

**Published:** 2023-01-28

**Authors:** Asad Ullah, Omer Saeed, Nabin Raj Karki, Mya Goodbee, Abdul Qahar Khan Yasinzai, Abdul Waheed, Saleh Heneidi, Anish Thomas, Nagla Abdel Karim, Joyce Johnson, Jaydira Del Rivero, Jaffar Khan

**Affiliations:** 1 Department of Pathology, Vanderbilt University Medical Center, Nashville, TN 37232, USA; 2 Department of Pathology, Indiana School of Medicine, Indiana University, Indianapolis, IN 46202, USA; 3 Department of Hematology and Oncology, University of South Alabama, Mobile, AL 36688, USA; 4 Medical College of Georgia, Augusta University, Augusta, GA 30912, USA; 5 Department of Medicine, Bolan Medical College, Quetta 83700, Pakistan; 6 Department of Surgery, San Joaquin General Hospital, San Joaquin, CA 95231, USA; 7 Department of Molecular Pathology, Cedars Sinai Medical Center, Los Angeles, CA 90048, USA; 8 Department of Hematology and Oncology, National Institute of Health (NIH), Bethesda, MA 20814, USA; 9 Department of Hematology and Oncology, Inova Schar Cancer Institute, Fairfax, VA 22031, USA

**Keywords:** combined small cell, lung cancer, histology, chemotherapy, molecular changes

## Abstract

Background: Primary lung cancer is the most common cause of cancer-related mortality in the United States (US). Approximately 90% of lung cancers are associated with smoking and the use of other tobacco products. Based on histology, lung cancers are divided into small-cell lung carcinomas (SCLCs) and non-small-cell lung carcinomas (NSCLCs). Most SCLCs are of the pure subtype, while the rare combined SCLCs contain elements of both small-cell and non-small-cell morphologies. This study sought to evaluate the demographics, clinical factors, molecular abnormalities, treatment approaches, and survival outcomes with combined SCLC and NSCLCs. Materials and Methods: Data on 2126 combined SCLC patients was extracted from the Surveillance Epidemiology and End Result (SEER) database from 2000 to 2018. Data extracted for analyses included age, sex, race, tumor size, tumor location, metastasis status, stage at diagnosis, treatment received, and treatment outcomes. Multivariate analysis was performed using Statistical Product and Service Solutions (SPSS) software. Results: The patients had a median age of 68 years; 43.9% of the patients were female and 56.1% were male; 84.5% were White and 11.7% were African Americans. The majority of patients had a poorly differentiated disease at 29.6%; 17% were undifferentiated, 3.2% were moderately differentiated, and 0.8% were well differentiated. Chemotherapy was the most common treatment modality (45.3%); 17% underwent surgery only, 10.3% underwent surgery followed by adjuvant chemotherapy, and 10% underwent radiation after surgery. Five-year cancer-specific survival was 15.2% with surgery alone, and combined surgery and chemotherapy provided the highest percentages (38.3% and 34.7%, respectively). Females had significantly higher 1- and 5-year cancer-specific survival rates compared to males (59.3% and 29.9% vs. 48.0% and 23.7, respectively; *p* < 0.001). Well-differentiated tumors had significantly higher survival compared to other gradings (*p* < 0.001). Survival decreased as tumor staging moved distally from localized to regional to distant (*p* < 0.001). Metastasis to bone, liver, brain, and lung significantly decreased survival in comparison to patients who did not have any metastasis (*p* < 0.001). Females had significantly shorter survival compared to their counterparts when metastasis was to the bone, brain, or liver (*p* < 0.001). Multivariate analysis identified male sex (Hazard Ratio (HR) = 1.2), undifferentiated grade (HR = 1.9), regional extent of disease (HR = 1.7), distant extent of disease (HR = 3.7), and metastasis to liver (HR = 3.5) as variables associated with worse survival. Conclusion: Combined SCLC is overall very rare. However, the frequency of presentation with combined SCLC is on the rise, in part due to improvements in diagnostic techniques. Despite advances in therapies, treating combined SCLC is challenging, and novel therapies are not utilized, owing to low rates of targetable mutations. Combined SCLC has higher survival rates if well differentiated.

## 1. Introduction

Lung cancer is the leading cause of cancer-related mortality in the United States of America. Despite advances in screening and treatment, lung cancer commonly presents late in the disease course, generally resulting in a poor prognosis. Risk factors include both genetic and environmental factors, with tobacco smoking being a well-known environmental exposure. [1,2]. Smoking increases the risk of both small-cell lung cancer (SCLC) and non-small-cell lung cancer (NSCLC), the two major histological types of lung cancer. The major subtypes of NSCLC are adenocarcinomas, squamous cell carcinomas, and large-cell carcinomas, whereas SCLC, which is a neuroendocrine tumor originating from the lung, can occur as either pure or combined subtypes [3]. In the combined cell subtype, SCLC coexists with at least 10% of large-cell carcinomas or any amount of adenocarcinoma, squamous cell carcinoma, or spindle cell carcinoma in the same contiguous tumor. Because the metastatic disease predominantly contains SCLC, and this does not correlate with the amount of SCLC component in the primary tumor, combined SCLC is approached more like standard SCLC. Combined SCLC should be distinguished from multiple primary lung cancers (MPLCs), which comprise >1 pathologically distinct primary tumor separated in space (different sites) or time.

SCLC accounts for approximately 13–20% of all lung cancer diagnoses, and combined SCLC accounts for approximately 5–28% of SCLC diagnoses [4,5]. Although relatively rare, the incidence of combined SCLC has shown an increasing trend. The classic paradigm has been that the clinicopathological features of combined SCLC are intermediate between those of SCLC and NSCLC, and the therapeutic approach mirrors that of standard SCLC treatment. However, combined SCLC remains poorly characterized owing to the rarity and inconsistent use of a hybrid of SCLC and NSCLC approaches.

## 2. Materials and Methods

Data on 2126 combined SCLC patients was extracted from the Surveillance Epidemiology and End Result (SEER) database from 2000 to 2018. Data extracted includes demographic data, including age, sex, and race, and clinical data including primary tumor location, histologic grading, tumor size, staging, treatment modality (surgery, radiation, and/or chemotherapy), and overall survival. The categories for each variable were limited to those available in the SEER database. Data was exported to Statistical Product and Service Solutions (SPSS). Exclusion criteria included patients who did not have microscopic confirmation of SCLC and patients whose death was confirmed through autopsy or death certificate only. Endpoints that were examined included overall survival, mortality, and cancer-specific survival rates at 1, 2, 3, 4 and 5 years. Multivariate analysis was performed to determine which factors affected survival. Kaplan–Meier survival curves were created using International Business Machine (IBM) SPSS^®^v28.0.0.0(190) software. Univariate analysis was completed to identify significant factors for multivariate analysis (Cox regression model) with a *p*-value set at 0.25. Cox regression analysis was utilized to calculate the hazard ratio (HR) for various independent factors affecting mortality. For this, statistical significance was considered at *p* < 0.05.

## 3. Results

### 3.1. Demographic Characteristics

Of the 2126 patients in our cohort, 100% were adults with a mean age of 68 years old. The majority of patients were male (56.1%) and White (84.5%). African Americans, Asian or Pacific Islanders, and American Indians/Alaskan Natives made up the remainder of the cohort (11.7%, 3.1%, and 0.7%, respectively). Only one patient’s race was unknown (0.04%) (Table 1).

### 3.2. Tumor Characteristics

For our 2126 patients, the staging was divided into four categories: localized, regional, distant, and unstaged/unknown. Localized meant that the tumor was limited to the organ of origin and there was no spread beyond the organ of origin; there was infiltration past the basement membrane of the epithelium into the stroma of the organ. Regional metastasis meant that the tumor extended beyond the limits of the organ of origin (subcategorized into: regional direct extension, regional to lymph nodes, regional to both by direct extension and lymph nodes, and regional, not otherwise specified). Distant spread meant a tumor which has spread to the areas of the body that are distant or remote from the primary tumor, and/or distant metastasis comprising tumor cells which have broken away from the primary tumor, and the tumor has travelled to the other parts of the body. Unknown stage refers to cases for which there is no sufficient evidence available to adequately assign stage (https://training.seer.cancer.gov/staging/systems/summary/regionalized.html, accessed on 17 January 2023). Most of the tumors were staged (91.7%). Of those staged, most of the tumors were staged as distant (51.5%), 27.1% of tumors were staged as regional, and 13.1% were localized. In the majority of cases, the tumor size was 0–3 cm (*n* = 460 (21.6%)), followed by 3.1–5 cm in *n* = 272 (12.8%) and 5.1–7 cm in *n* = 155 (7.3%), and the tumor size was > 7 cm in *n* = 175 (8.2%) of cases (Table 2).

### 3.3. Lymph Node Status and Metastasis at the Time of Diagnosis

In total, 758 patients of the entire cohort had a known lymph node status (35.7%). Of the 758 patients, 464 patients had positive lymph node(s) (21.8%). In total, 537 patients had distant metastasis, with the majority to the liver (6.9%). Bone, brain, and lung metastases follow closely after (6.7%, 6.1%, and 5.5%, respectively) (Table 3).

### 3.4. Treatment Characteristics

Overall, 45.3% underwent chemotherapy and 17% underwent surgery; 10.3% of patients had a combination of surgery and chemotherapy. Radiation could be given prior to surgery, after surgery, or before and after surgery. However, the majority of patients who received radiation had it done after surgery (10.0%). A total of 1504 deaths were attributed to combined SCLC (70.7%) (Table 4).

### 3.5. Survival Characteristics by Treatment Modality

The overall 5-year survival rate for all 2126 patients was 12.4% with a 95% confidence interval (C.I. 95%, 10.7–14.3), and the cause-specific survival was 15.2% (C.I. 95%, 13.2–17.3). Patients who underwent only surgery had the highest 5-year survival at 38.3%. The 5-year survival with chemotherapy was 15.2%, and with radiation therapy the 5-year survival was 22.2%. A combination of surgery and chemotherapy had a similar 5-year survival rate of 34.7% (Table 5).

### 3.6. Survival by Gender and Race

**Figure 1:**
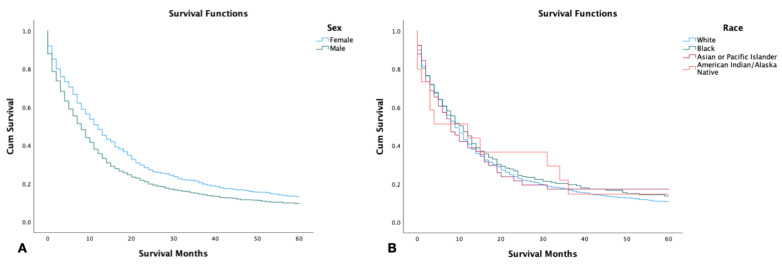
Kaplan–Meier survival graph of 2126 patients with combined small-cell lung cancer from SEER database by (2000–2018). (**A**) Survival by sex; (**B**) survival by race. Log rank (Mantel–Cox) analysis (**χ^2^**) performed on each independent variable. Sex was found to be a significant independent variable with *p <* 0.001, while race was not (*p* = 0.8).

Females had a significantly higher 5-year survival rate in comparison to their male counterparts (29.9%, 23.7%, respectively) (*p* < 0.001). American Indians, Asians, and Pacific Islanders had the highest 5-year survival at 42.0%, followed by African Americans and Whites (31.4%, and 24.5%, respectively). However, there was no significant difference in survival time between the different racial groups (*p* = 0.8) (Figure 1).

### 3.7. Survival by Tumor Size, Grading, and Stage

**Figure 2:**
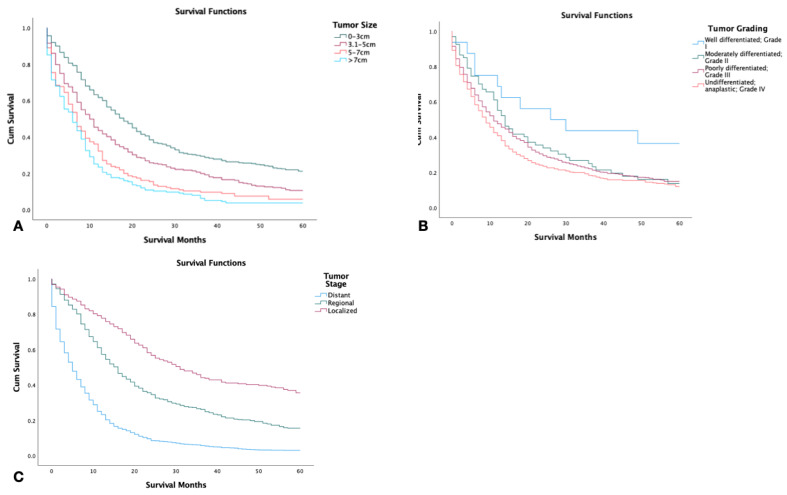
Kaplan–Meier survival graphs of 2126 patients with combined small-cell lung cancer from the surveillance epidemiology and end result (SEER) database by (**A**) tumor size, (**B**) tumor grade, (**C**) tumor stage. Log rank (Mantel–Cox) analysis (**χ^2^**) performed on each independent variable (*p* < 0.001).

Tumor size was found to have an inverse relationship with survival. Patients with tumors smaller than 3 cm displayed the highest 5-year survival, while patients with tumors larger than 7 cm had the lowest (*p* < 0.001) (Figure 2A). Patients with well-differentiated tumor grading had a significantly higher 5-year survival in comparison to patients with other tumor types including moderately differentiated, poorly differentiated, and undifferentiated (*p* < 0.001) (Figure 2B). Out of the 2126 patients, those with localized tumors had significantly higher survival in comparison to those with regional or distant staging (*p* < 0.001) (Figure 2C).

### 3.8. Survival with Metastasis to Liver, Bone, Brain, and Lung

**Figure 3:**
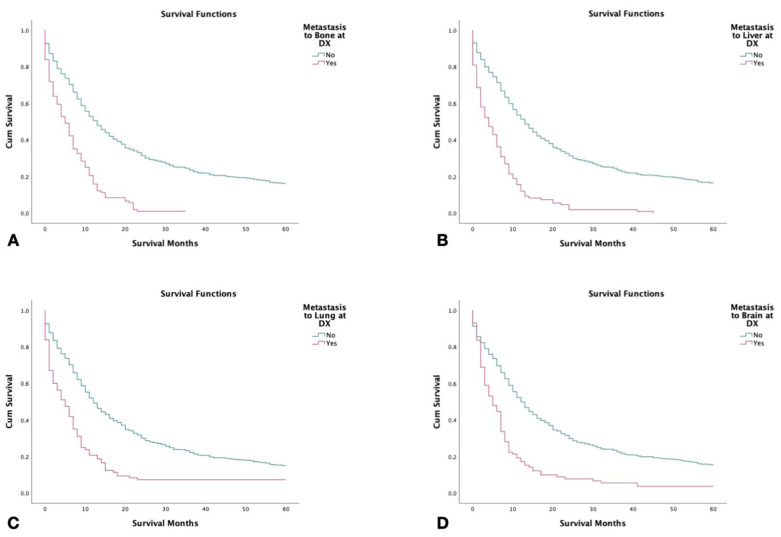
Kaplan–Meier survival curves of 2126 patients with combined small-cell lung cancer from SEER database by metastasis to liver at the time of diagnosis (2000–2018). (**A**) Survival by metastasis to the bone at the time of diagnosis. (**B**) Survival by metastasis to the liver at the time of diagnosis. (**C**) Survival by metastasis/spread to the lungs at the time of diagnosis. (**D**) Survival by metastasis to the brain at the time of diagnosis. Log rank (Mantel–Cox) analysis (**χ^2^**) performed on each independent variable (*p* < 0.001).

**Figure 4:**
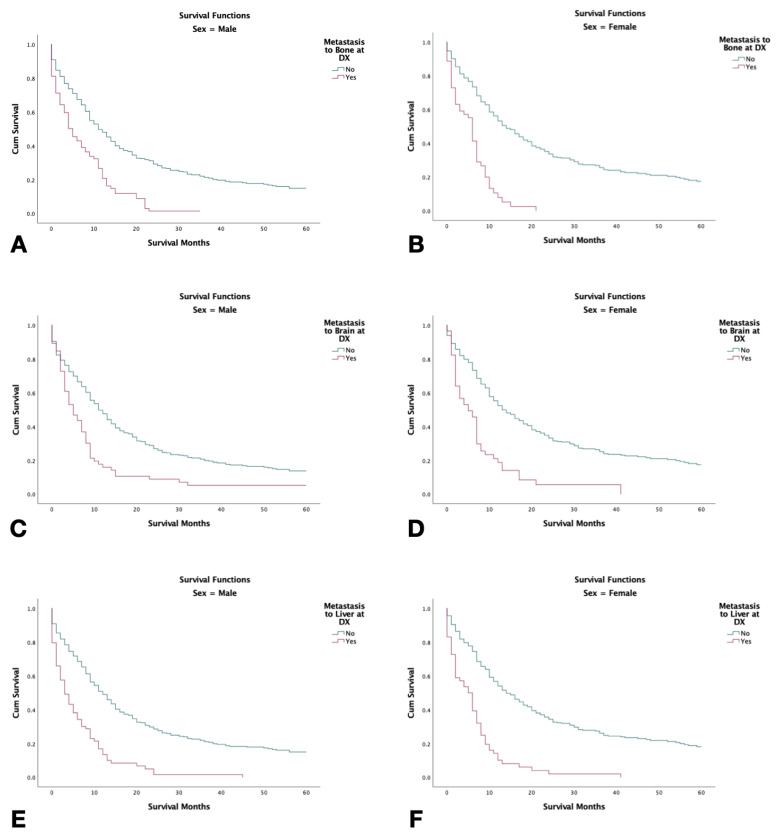
Kaplan–Meier survival graphs of 2126 combined small-cell lung cancer patients with metastasis at the time of diagnosis stratified by sex with (2000–2018). (**A**,**B**) Survival in males and females with metastasis to bone. (**C**,**D**) Survival in males and females with metastasis to the brain. (**E**,**F**): Survival in males and females, respectively, with metastasis to the liver. Log rank (Mantel–Cox) analysis (**χ^2^**) performed on each independent variable (*p* < 0.001).

Patients with no metastatic disease had significantly higher survival compared to patients with metastasis to lung, bone, brain, or liver at the time of diagnosis (*p* < 0.001). Patients with metastasis to the bone had the shortest 5-year survival with patients with metastasis to the liver closely following (Figure 3). In patients who had metastasis to the bone, brain, or liver, females were found to have significantly shorter survival in comparison to males (*p* < 0.001) (Figure 4).

### 3.9. Multivariate Analysis

To determine which factors to utilize for multivariate analysis, univariate analysis was performed on multiple variables, including sex, race, tumor grade, tumor stage, tumor size, and metastasis to liver, brain, bone, and lung. The significance for this was set at *p* < 0.25. In regard to the multivariate analysis, a significance of *p* < 0.05 was set. Cox regression analysis determined that male gender (hazard ratio (HR), *p*-value, HR 1.2, *p* < 0.001), undifferentiated tumor grade (HR 1.9, *p* = 0.04), regional metastasis (HR 1.7, *p* < 0.001), distant metastasis (HR 3.7, *p* < 0.001), and specifically liver metastasis at the time of diagnosis (HR 3.5, (*p* = 0.007) were associated with worse survival (Table 6).

## 4. Discussion

Combined SCLC is relatively rare. Small-cell lung cancers comprise 13–20% of all lung cancers, and combined SCLC makes up 5–28% of SCLC cases [5]. However, the incidence of combined SCLC may be on the rise owing to improved diagnostics and lung cancer screening programs. Moreover, the rates of diagnosis of combined SCLC are higher when lung resection samples are analyzed as opposed to biopsy specimens. By definition, combined SCLC comprising area(s) of small-cell histology are admixed with any of non-small-cell histology such as adenocarcinoma, squamous cell carcinoma, large-cell carcinoma, large-cell neuroendocrine carcinoma, spindle cell carcinoma, or giant cell carcinoma in the same tumor specimen. Interestingly, combined SCLC can also be a part of MPLC, either as synchronous or metachronous primary tumors. While MPLC comprises any ≥2 primary lung cancer histological subtypes (including combined SCLC) that are discrete, combined SCLC is a mixture of SCLC and ≥1 NSCLC histology in the same tumor.

Combined SCLC is a disease affecting the elderly population. The median age of presentation of combined SCLC at 66 years in our study is similar to another published case series [6,7]. Our study included male and female patients in a ratio of 3:5, while varying gender distributions have been reported. One study of 35 cases showed 43% males, where another study of 97 cases showed 82.5% cases, likely a result of different patterns of smoking exposure [7,8]. White predominance has been noted elsewhere [7]. However, the extent to which this represents differences in population composition, occupational exposure, and access issues remains unknown. Most patients with combined SCLC have a history of smoking. Moreover, 88% of our patients had a history of smoking, compared to 95% in a larger historical case series [9].

The right lung was involved in 71% of cases. A Chinese study reported a longer survival in right-sided combined SCLC compared to the left sided ones [5]. Compared to pure SCLC, combined SCLC is more peripherally located, i.e., about half of combined SCLC are peripherally located, and pleural effusion is more commonly seen. Approximately 70% of cases are diagnosed at a limited stage, with approximately 30% at stage I-II based on tumor, node, metastasis (TNM) (seventh edition) [5]. The most common non-small-cell partner was adenocarcinoma, followed by squamous carcinoma. This is in stark contrast to other studies where the predominant partner was large cells (47–80%) [6,7,8,9]. EGFR mutations have been reported in combined SCLC at a frequency slightly higher than that in pure-SCLC [10,11]. One study reported that approximately 10% of patients with SCLC have brain metastases at the time of diagnosis, which is more than what we observed in our study [12].

Compared to pure SCLC, combined SCLC is thought to derive more benefit from surgery, less benefit from chemotherapy and radiotherapy, and more benefit from targeted therapy, especially anti-EGFR tyrosine kinase inhibitors [5]. However, approximately one-half of our patients received chemotherapy only, and one-tenth received surgery with adjuvant chemotherapy. Only one-fifth of our patients underwent surgery only. EGFR mutations are rare in SCLC but may be slightly more common in combined SCLC, especially in cases of adenocarcinoma, and reports of response to anti-EGFR TKIs are available [10]. Guidelines specified for combined SCLC treatment are lacking, and the recommendation is to treat combined SCLC along with the SCLC guidelines. As per the national comprehensive cancer network (NCCN), T1-T2N0M0 receive surgery for SCLC, while stage IA, IB, IIA, IIB, and IIIA undergo surgery [13].

Overall survival was higher in combined SCLC than in pure SCLC (15 months vs. 10.8 months, *p* = 0.035), but this difference disappeared in patients who did not undergo surgery [6]. No difference in survival was observed in a Chinese study [14]. In another Chinese study, among those with limited disease, 5-year overall survival was 48.9% post-surgery compared to 36.6% among those who did not undergo surgery [15]. Thus, surgery should be the key modality for patients who are candidates for radical resection [16]. Good prognostic factors include surgical resectability, limited disease extent, good performance status, right lung location, and central site, combination with adenocarcinoma or spindle cell carcinoma, and normal C-reactive protein levels [5]. Large cell histology, poor performance status, extensive spread, elevated serum lactate dehydrogenase, elevated neuron-specific enolase levels, and elevated neutrophil-to-lymphocyte ratio were associated with a worse prognosis [14].

### Genomic and Treatment Landscape of Combined Small-Cell Lung Cancer

A recent study by a Chinese group suggested that combined SCLC components are derived from the same pluripotent clonal stem cells and share initial driver mutations with subsequent genomic alterations [17]. However, pulmonary combined SCLC and combined large-cell neuroendocrine carcinoma (combined LCNEC) were noted to possess different interacting driver genes, as exemplified by the lower incidence of EGFR mutations in combined SCLC than in combined LCNEC (5% vs. 25.7%, *p* = 0.004) [18]. These two tumor types also differed in terms of clinical phenotype, and combined LCNEC had better outcomes than the combined SCLC. TP53 and RB1 were the most altered genes in 12 patients (83.3% and 66.7%, respectively). Another case series of 13 combined SCLC patients showed KRAS G12C (2/13), PI3KCA (2/13), and EGFR (1/13) alterations, which are potentially targetable [17]. Other frequently noted genes in combined SCLC are PTEN, TERT, ARID1B, SDHA, NF1, NOTCH2, NOTCH1, ALK, FGFR4, FGFR1, SOX2, NOTCH3, KMT2D, FAT1, and FAM135B [18,19]. Although rare, case reports suggest that the presence of EGFR and ALK alterations may predict benefits from targeted agents [11,20]. Anlotinib (an oral multikinase tyrosine kinase inhibitor, approved in China for third-line therapy for SCLC) achieved partial response in a patient with combined SCLC [21]. The rarity of combined SCLC makes conducting larger studies tailored to a specific phenotype/genotype very challenging. There are 116 active phase 3/4 trials for small-cell lung carcinomas (clinicaltrials.gov, accessed on February 13, 2022). While some of these trials may allow for the enrollment of combined SCLC patients on a case-by-case basis, only one phase 2 trial has combined SCLC as a prespecified enrollment category [22]. The following paragraph lays out the standard of care and outlines themes for future investigations of standard-SCLC, which may be extrapolated to combined SCLC.

The treatment paradigm for combined SCLCs is based on pure/standard SCLCs. Surgery is the default option for very small tumors (cT1N0M0). Concurrent platinum doublet chemoradiotherapy has long been the standard treatment for limited-stage SCLC, with demonstrable overall survival exceeding 2 years. Prophylactic cranial irradiation is generally recommended after curative intent chemoradiation for limited-stage SCLC if cranial imaging results are negative. Four to six cycles of platinum doublet therapy (carboplatin and etoposide) have long been the standard treatment for advanced/metastatic extensive-stage SCLC. Atezolizumab and durvalumab are now recommended to be added for first-line treatment based on IMPOWER-133 and CASPIAN study, respectively [23,24]. Both studies showed improved survival and progression-free survival as well as acceptable adverse event profile. Topotecan, approved in 2007 and more recently, lurbinectedin, approved in 2020 are the only approved second-line chemotherapies [25]. There is ongoing interest in the utilization of different immunotherapies and combinations with chemotherapy, immunotherapy, radiotherapy, or surgery [26]. Targeting the DNA repair pathway (veliparib for PARP SLFN11 high tumors), genomic alterations (e.g., pazopanib for FGFR1-amplified tumors), antigens expressed on cancer cells with antibody-drug conjugates (for example, rovalpituzumab tesirine for DLL3 expressed in SCLC cells), and genomic instability (e.g., checkpoint inhibitors) are areas of active research [27,28]. In a recent study, immune checkpoint inhibitors (ICIs) were found to prolong progression-free survival and overall survival when paired with chemotherapy in patients with advanced SCLC. This combination was found to be superior to conventional chemotherapy. However, there were higher amounts of side effects including fatigue, rashes, diarrhea, and increased liver enzymes in the ICIs combined with chemotherapy group [29].

## 5. Limitations

Limitations of our study include the limited dataset of the retrospective registry, such as the margin status of the resected tumor. Information on the non-small-cell component of the tumor was not available. Lung cancers are closely linked to smoking, the smoking data in these patients were not available in the database. Furthermore, the dataset lacks information on genetic mutations and the type of chemotherapy received at the time of management. An organized, national, and/or international effort is needed to gather and sequence newly identified patients of combined small-cell lung carcinoma for appropriate genetic sequencing and future personalized therapeutic approaches.

## 6. Conclusions

Combined SCLC is rare, but the frequency of diagnosis of this entity is on the rise owing to improvements in diagnostic techniques. In our study, we found that male gender, distant metastasis, and tumor size greater than 7 cm were associated with worse outcomes., while in metastatic disease, female gender had worse outcomes compared to the male gender. The presentation of combined SCLC is more common in patients with a history of tobacco smoking, highlighting the role of smoking cessation in patients who present with combined SCLC at diagnosis. The treatment approaches mirror those of standard (pure) SCLCs. Despite advances in therapies for lung cancers, the treatment of combined SCLC has not undergone a major overhaul, and controversy exists regarding the use of new therapies beyond traditional surgery, chemotherapy, and radiation protocols.

## Figures and Tables

**Table 1:** Demographics and Clinical Profile of 2126 Patients with Combined Small-Cell Lung Cancer from Surveillance Epidemiology and End Result (SEER) Database (2000–2018).

	Overall
*n* (%)	2126 (100.0)
Age (Mean ± SD)	68.2 ± 10.1
Gender	
Male	1192 (56.1)
Female	934 (43.9)
Race *n* (%)	
White	1796 (84.5)
African American	248 (11.7)
Asian or Pacific Islander	66 (3.1)
American Indian/Alaskan Native	15 (0.7)
Unknown	1 (0.04)

**Table 2:** Tumor Characteristics of 2126 Patients with Combined Small-Cell Lung Cancer from Surveillance Epidemiology and End Result (SEER) Database (2000–2018).

	Overall
*n* (%)	2126 (100.0)
Stage *n* (%)	
Localized	279 (13.1)
Regional	576 (27.1)
Distant	1094 (51.5)
Unstaged/Unknown	177 (8.3)
Tumor Size *n* (%)	
0–3 cm	460 (21.6)
3.1–5 cm	272 (12.8)
5.1–7 cm	155 (7.3)
>7 cm	175 (8.2)

**Table 3:** Lymph Node Status and Distant Metastasis Location of 2126 Patients with Combined Small-Cell Lung Cancer at the Time of Diagnosis from Surveillance Epidemiology and End Result (SEER) Database (2000–2018).

Lymph Node Status	
Known *n* (%)	758 (35.7)
Positive Lymph Node *n* (%)	464 (21.8)
Negative Lymph Node *n* (%)	294 (13.8)
Unknown *n* (%)	1368 (64.3)
Distant Metastasis	
Liver Metastasis *n* (%)	147 (6.9)
Bone Metastasis *n* (%)	143 (6.7)
Brain Metastasis *n* (%)	130 (6.1)
Lung Metastasis *n* (%)	117 (5.5)

**Table 4:** Treatment Characteristics of 2126 Patients with Combined Small-Cell Lung Cancer from Surveillance Epidemiology and End Result (SEER) Database (2000–2018).

	Overall
*n* (%)	2126 (100.0)
Treatment *n* (%)	
Surgery Only	362 (17.0)
Radiation Prior to Surgery	9 (0.4)
Radiation After Surgery	212 (10.0)
Radiation Before and After Surgery	3 (0.1)
Chemotherapy	964 (45.3)
Combination of Surgery and Chemotherapy	219 (10.3)
Unknown	357 (16.8)
Overall Mortality *n* (%)	1841 (86.6)
Cancer-Specific Mortality *n* (%)	1504 (70.7)

**Table 5:** Survival data of 2126 Patients with Combined Small-Cell Lung Cancer from the Surveillance Epidemiology and End Result (SEER) Database (2000–2018).

Survival	Overall % (95% C.I)	Cause- Specific % (95% C.I)	Surgery % (95% C.I)	Radiation % (95% C.I)	Chemotherapy % (95% C.I)	Combined Surgery and Chemotherapy % (95% C.I)
1 year	44.2 (41.6–46.7)	46.4 (43.7–48.9)	76.9 (72.1–81.0)	60.4 (53.2–66.8)	55 (51.7–58.2)	80.8 (74.8–85.5)
2 years	24.9 (22.7–27.2)	26.9 (24.5–29.3)	57.8 (52.3–62.9)	37.8 (31.0–44.6)	29.9 (26.9–33.0	55.8 (48.7–62.3)
3 years	18.6 (16.6–20.7)	21.0 (18.8–23.2)	49.5 (43.9–54.8)	29.7 (23.3–36.4)	22.3 (19.5–25.2)	46.4 (39.3–53.3)
4 years	14.5 (12.7–16.4)	17.2 (15.1–19.3)	43.1 (37.5–48.5)	25.9 (19.8–32.5)	17.5 (14.9–20.2)	40.3 (33.2–47.2)
5 years	12.4 (10.7–14.3)	15.2 (13.2–17.3)	38.3 (32.7–43.8)	22.2 (16.3–28.7)	15.2 (12.7–17.9)	34.7 (27.7–41.7)

**Table 6:** Multivariate Analysis of Independent Factors Affecting 2126 Patients with Combined Small-Cell Lung Cancer from Surveillance Epidemiology and End Result (SEER) Database (2000–2018).

Variables	Univariate Analysis	Multivariate Analysis	
	ANOVA F Value (*p*-Value)	Hazard Ratio (*p*-Value)	Confidence Interval
Male Sex	25.1 (<0.001)	1.2 (<0.001)	1.1–1.3
Undifferentiated Grade	14.9 (<0.001)	1.9 (0.042)	1.0–3.6
Regional Extent of Disease	267.5 (<0.001)	1.7 (<0.001)	1.4–2.1
Distant Extent of Disease		3.7 (<0.001)	3.1–4.4
Liver Metastasis	25.9 (<0.001)	3.5 (0.007)	1.4–8.7

## Data Availability

Not applicable.
